# The diagnostic status of chronic kidney disease in a real-world database in Japan: CHECK-CKD

**DOI:** 10.1007/s10157-025-02682-z

**Published:** 2025-05-27

**Authors:** Toshiki Moriyama, Keigo Kanafuri, Mayu Kanno, Koji Niibe, Sachiko Nago, Ichiro Fukuoka, Yasuhisa Fukunaga, Issei Doi, Masaru Kawashima

**Affiliations:** 1 Health and Counseling Center, Osaka University, 1-17, Machikaneyama-cho, Toyonaka-shi, Osaka 560-0043 Japan; 2 Medical Affairs, Ono Pharmaceutical Co., Ltd, 8-2, Kyutaromachi 1-chome, Chuo-ku, Osaka 541-8564 Japan; 3 Digital Technology, Ono Pharmaceutical Co., Ltd, 8-2, Kyutaromachi 1-chome, Chuo-ku, Osaka 541-8564 Japan

**Keywords:** CKD diagnosis rates, Chronic kidney disease, Japan, Real-world data, Renal function tests, Nephrology

## Abstract

**Background:**

The clinical practice guidelines for chronic kidney disease (CKD) in 2018 and the launch of the first therapeutic agent in 2021 are expected to have improved CKD management in Japan. However, the reality of CKD diagnosis in this environment is poorly understood. Here, we conducted a retrospective observational study.

**Methods:**

We investigated the changes in CKD diagnosis rates, the characteristics of diagnosed cases, and the prognostic impact of the timing of diagnosis by using a database of administrative claims and medical checkups from 2014 to 2023 (DeSC Healthcare Inc.™) for patients with a potential risk of CKD (eGFR <60 mL/min/1.73 m^2^ and/or urine protein qualitative test result of ≥1+).

**Results:**

We extracted 287,999 patients who newly met the diagnostic criteria for CKD at a medical checkup. The rate of new CKD diagnosis remained ~ 3% until 2021. Factors associated with CKD diagnosis included blood/urine tests at a medical institution (odds ratio [OR] 4.11, 95% confidence interval [CI] 3.92–4.31; OR 5.02, 95% CI 4.82–5.22) and presence of comorbidities: anemia (OR 2.30; 95% CI 2.16–2.45), heart failure (OR 1.87; 95% CI 1.76–1.98), and diabetes (OR 1.84; 95% CI 1.76–1.91). The incidence of cardiorenal-related events at 36 months after the date when patients newly met the CKD diagnostic criteria was 4.5% and 12.4% for those diagnosed at stage 3a and 3b, respectively.

**Conclusions:**

The prevalence of CKD diagnosis was low and renal function tests were infrequently performed. Periodic blood/urine tests may help clinicians to detect CKD in an early phase.

**Study registration:**

UMIN000052393

**Supplementary Information:**

The online version contains supplementary material available at 10.1007/s10157-025-02682-z.

## Introduction

Chronic kidney disease (CKD) is a disease characterized by persistent renal impairment and renal dysfunction. CKD often progresses to end-stage kidney disease, which necessitates dialysis and kidney transplantation [1]. CKD is also associated with cerebrovascular disease and death [2]. For these reasons, early therapeutic intervention is important to delay disease progression [3, 4]. However, since CKD is usually asymptomatic in its early stages, its diagnosis and treatment are frequently delayed [2]. The importance of diagnosing CKD has been reconsidered.

The prevalence of CKD in Japan is steadily increasing due to the advanced aging of the population and increasing prevalence of CKD-related risk factors, particularly type 2 diabetes [5–7]. In 2005, about 13.3 million people, or 13% of the adult population in Japan, were estimated to have CKD and in 2015, about 14.8 million people were estimated to have CKD, but only about 393,000 patients with CKD were receiving continuous treatment in 2017 [3, 8, 9]. Therefore, we can assume that there is a large population of people living with undiagnosed and untreated CKD in Japan.

In the last decade, several efforts have been made to promote the diagnosis and treatment of CKD in Japan. For example, the first Study Committee on Kidney Disease Countermeasures was held, and the CKD Clinical Practice Guidelines were revised in 2018 [3, 10, 11]. However, the clinical guidelines are under-utilized among general practitioners [12, 13]. In addition to the cornerstone medications (angiotensin converting enzyme inhibitors and angiotensin II receptor blockers) for which renoprotective effects were revealed in patients with type 2 diabetes [14], several recent trials showed the renoprotective effects of sodium-glucose cotransporter-2 (SGLT2) inhibitors [15–17]. In Japan, an SGLT2 inhibitor was launched for the indication of CKD in August 2021. These changes in the treatment environment were also expected to have influenced the management of CKD. However, no report has clarified the status of CKD diagnosis following these changes. Therefore, we sought to investigate the changes in the CKD diagnosis rate over time, the factors that influenced the diagnosis of CKD, and the prognostic impact of the timing of CKD diagnosis in a real-world setting in Japan.

## Methods

### Data source

We performed a cross-sectional study of the DeSC database (DeSC Healthcare Inc.™, Chiyoda, Tokyo, Japan) containing claims data from approximately 18.8 million residents in Japan who are covered by Society-Managed Health Insurance, National Health Insurance managed by municipalities, and the Medical Care System for the Elderly, for the period between April 2014 and March 2023 [18]. The database includes data collected at annual medical checkups. The DeSC database tracks patients if they receive diagnoses, treatments, or procedures at different medical institutions until a change in health insurance association, because it anonymizes and collates patient information from both inpatient and outpatient services. The study was registered on the University Hospital Medical Information Network Clinical Trial Registry (UMIN000052393).

### Patient eligibility criteria

Patients aged ≥18 years with medical records between April 2014 and March 2023 who were at potential risk of CKD were included. The index date for data extraction was defined as having eGFR <60 mL/min/1.73 m^2^ and/or a urine protein qualitative test result of ≥1+ (i.e., positive), and who met all of the following criteria prior to the index date: (i) records of eGFR ≥ 60 mL/min/1.73 m^2^, (ii) records of urine protein <1+ (i.e., negative), and (iii) no medical records of CKD. A subset of patients with CKD medical records after the index date was also identified. CKD was staged according to eGFR (in mL/min/1.73 m^2^) [4]. Diagnoses (including CKD, complications, and cardiorenal-related events) and procedures were identified using the International Classification of Diseases, 10th revision (ICD-10) and procedure codes listed in Table S1 (Online Resource 1).

### Study outcomes and data analysis

#### Proportion of patients diagnosed with CKD per year

Among eligible patients, the proportion of patients diagnosed with CKD was determined for each 1-year period (August–July) among those who (i) both underwent a medical checkup and met the CKD diagnostic criteria in each period if they had follow-up data for 6 months, (ii) had a recorded visit to the medical institution within 6 months after the medical checkup, and (iii) had no records of CKD before or on the date of the medical checkup.

#### Characteristics of patients diagnosed with CKD

Among eligible patients, we identified patients with and without diagnosis of CKD. Patients with CKD were matched to those without CKD by timepoint in each observation period, without replacement, at a ratio of 1:1 (Table S2 [Online Resource 1]). Patients without CKD were randomly selected from the at-risk population from the same time-period. A total of 22,207 patients were randomly extracted for each group. Their medical records were retrieved for a period of 12 months before, and including, the timepoint used for matching. Multivariable logistic regression was performed to determine the odds ratios (ORs) with 95% confidence intervals (CIs) for CKD diagnosis. Covariates included demographic and clinical characteristics. The most recent data recorded prior to the matching timepoint were used.

#### Percentage and characteristics of patients who underwent blood/urine tests in 2021

Among eligible patients who underwent a medical checkup between August 2021 and July 2022, we determined the percentages of those with history of blood/urine tests at medical institutions within 6 months of the medical checkup, if they were followed up for 6 months and had a recorded visit to a medical institution within 6 months after the medical checkup. History of blood/urine tests was defined using relevant procedure codes. The patient characteristics were tabulated using the information retrieved from the medical records at, or within 12 months before, the medical checkup date.

#### Incidence of cardiorenal-related events according to the stage at CKD diagnosis and the annualized change in eGFR

Among eligible patients, the incidence of cardiorenal-related events in patients without a cardiorenal-related event before being newly diagnosed with CKD was compared by stage at CKD diagnosis using the Kaplan–Meier method, starting from the index date to an event, for patients classified as stage 3a or stage 3b at diagnosis. CKD stage was defined based on eGFR recorded up to 12 months before the new diagnosis of CKD. The incidence of cardiorenal-related events, among patients without such events prior to a new diagnosis, was also compared according to the annualized decline in eGFR.

## Results

### Patient characteristics

**Fig. 1:**
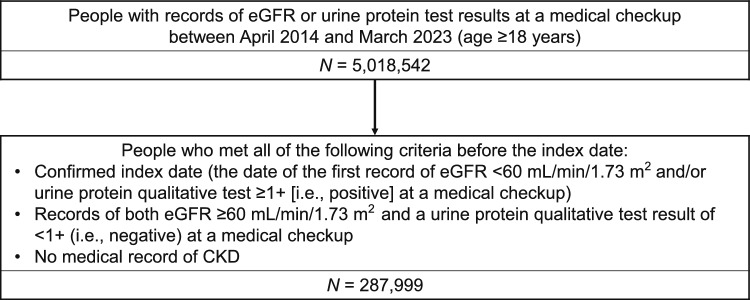
Patient disposition. *CKD* chronic kidney disease, *eGFR* estimated glomerular filtration rate

Of 5,018,542 patients with eGFR and/or urine protein test results recorded at medical checkups performed at ≥18 years of age, 287,999 were eligible for this study (Fig. 1). Their characteristics at the index date are summarized in Table 1.

**Table 1:** Patient characteristics^a^

Characteristic	Value (*N* = 287,999)
Sex	
Male	141,872 (49.3)
Female	146,127 (50.7)
Age (years)	
<65	129,983 (45.1)
65 to <75	103,403 (35.9)
≥75	54,613 (19.0)
Mean ± SD	62.9 ± 13.6
Median (Q1–Q3)	66.0 (53.0‒71.0)
BMI (kg/m^2^)	
<18.5	19,895 (6.9)
18.5 to <25.0	183,806 (63.9)
25.0 to <30.0	69,689 (24.2)
≥30.0	14,464 (5.0)
Unknown	145 (0.1)
Mean ± SD	23.38 ± 3.73
Median (Q1–Q3)	23.00 (20.80‒25.40)
History of smoking	
Yes	24,548 (12.3)
No	174,371 (87.7)
Unknown	89,080 (30.9)
Complications (in ≥5% of patients)	
Hypertension	114,125 (39.6)
Dyslipidemia	106,049 (36.8)
Diabetes	49,712 (17.3)
Hyperuricemia	20,095 (7.0)
Ischemic heart disease	21,854 (7.6)
Heart failure	19,769 (6.9)
Stroke	17,497 (6.1)
Anemia	15,711 (5.5)
Examination history	
Blood tests	138,849 (48.2)
Urine tests	70,568 (24.5)
Insurer type	
Health insurance	95,057 (33.0)
National Health Insurance	138,079 (47.9)
Medical insurance system for the elderly	54,863 (19.0)
eGFR (mL/min/1.73 m^2^)	
Stage 1: ≥90	14,539 (5.1)
Stage 2: ≥60 to <90	69,367 (24.3)
Stage 3a: ≥45 to <60	199,229 (69.8)
Stage 3b: ≥30 to <45	2071 (0.7)
Stage 4: ≥15 to <30	150 (0.1)
Stage 5: <15	243 (0.1)
Unknown	2400 (0.8)
Mean ± SD	63.06 ± 13.17
Median (Q1–Q3)	58.80 (56.56‒65.62)
Urine protein	
−	172,463 (60.0)
±	20,402 (7.1)
1+	83,542 (29.1)
2+	9519 (3.3)
3+	1470 (0.5)
4+	9 (0.0)
Unknown	594 (0.2)

Values are *n* (%) unless otherwise specified

*BMI* body mass index, *eGFR* estimated glomerular filtration rate, *Q* quartile, *SD* standard deviation

^a^Patient characteristics were retrieved from the medical records at, or within 12 months before, the index date

### CKD diagnosis rate by year

**Fig. 2:**
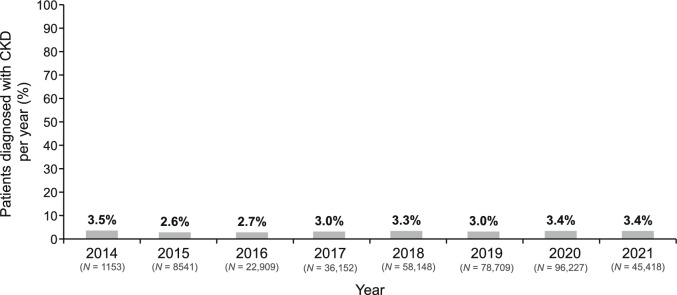
Percentage of patients diagnosed with CKD per annual period (August–July). *CKD* chronic kidney disease

The rate of new CKD diagnoses per year did not change over time, with rates of 3.0% in 2017 (when the Kidney Disease Review Committee was established), 3.3% in 2018 (when the CKD guidelines were revised), and 3.4% in 2021 (when an SGLT2 inhibitor for CKD was approved) (Fig. 2).

### Patient characteristics associated with CKD diagnosis

**Fig. 3:**
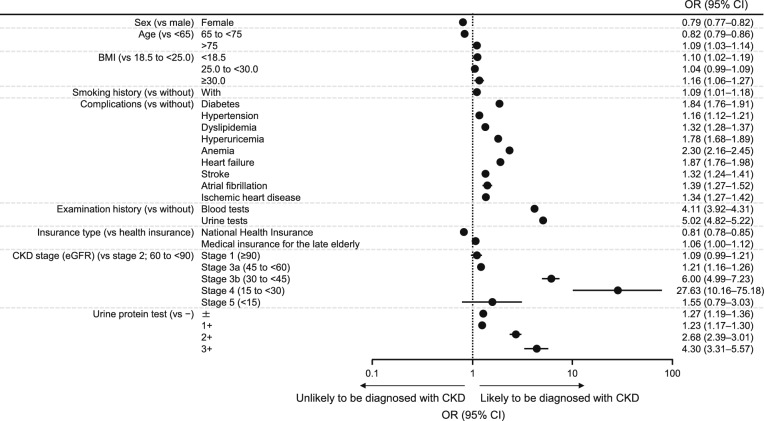
Likelihood of CKD diagnosis among timepoint-matched patients with and without confirmed CKD diagnosis. Patients with and without CKD were matched at the ratio of 1:1 without replacement at each timepoint of the observation period. Patients without CKD were randomly selected from the at-risk population from the period corresponding to patients with CKD. The characteristics of the patients were extracted and multivariable logistic regression was performed to determine the OR and 95% CI for CKD diagnosis, which was identified using relevant International Classification of Diseases, 10th revision, diagnostic or procedure codes. Characteristics of the matched cohorts are presented in Table S2 (Online Resource 1). The observation items included sex, age (years), BMI (kg/m^2^), smoking history, complications, blood test history, urinalysis history, category of insurer, department of consultation, region of the consultation facility, hospital category (number of beds), eGFR (mL/min/1.73 m^2^), and urinary protein. *BMI* body mass index, *CI* confidence interval, *CKD* chronic kidney disease, *eGFR* estimated glomerular filtration rate, *OR* odds ratio

Timepoint matching was used to match patients with and without CKD diagnosis (defined according to records of ICD-10 codes for CKD), and ORs with 95% CIs were calculated to identify factors associated with CKD diagnosis. The characteristics of the matched cohorts are summarized in Table S2 (Online Resource 1). Factors associated with the diagnosis of CKD included diabetes (OR 1.84; 95% CI 1.76–1.91), anemia (OR 2.30; 95% CI 2.16–2.45), heart failure (OR 1.87; 95% CI 1.76–1.98), and history of blood tests (OR 5.02; 95% CI 4.82–5.22) and urine tests (OR 4.11; 95% CI 3.92–4.31) at a medical institution checkup (Fig. 3). Male sex, age ≥75 years, hypertension, hyperuricemia, higher CKD stages, and positive results of urine protein test were also associated with diagnosis of CKD.

### Distribution of blood and urine tests according to the characteristics of patients satisfying CKD criteria in 2021

We determined how frequently blood and urine tests were performed at the medical institutions, within 6 months after the medical checkup for patients at risk for CKD, among 43,688 patients without history of CKD diagnosis who met the CKD criteria between August 2021 and July 2022. We chose this period because it is the latest year in which there was adequate follow-up of patients for the analysis. The characteristics of these patients are summarized in Tables S3 and S4 (Online Resource 1) for patients divided according to history of blood tests or urine tests, respectively. Overall, 18,912 (43.3%) and 9110 (20.9%) patients had histories of blood or urine tests at the medical institutions, respectively.

**Fig. 4:**
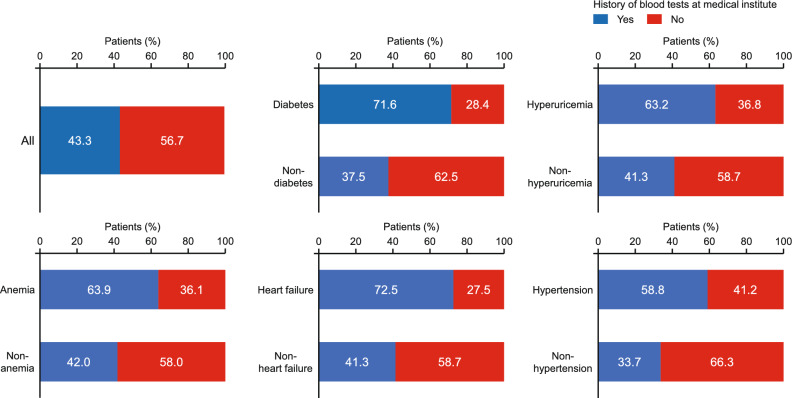
Proportions of patients with or without history of blood tests performed at a medical institution visit according to the presence and absence of common comorbidities (diabetes, hyperuricemia, anemia, heart failure, and hypertension)

**Fig. 5:**
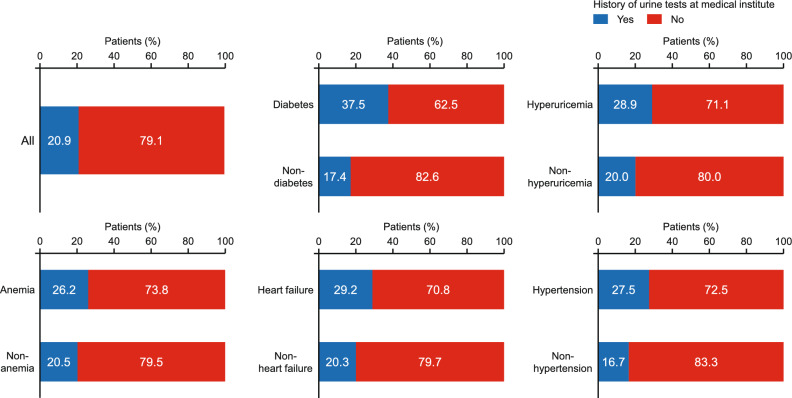
Proportions of patients with or without history of urine tests performed at a medical institution visit according to the presence and absence of common comorbidities (diabetes, hyperuricemia, anemia, heart failure, and hypertension)

We also investigated the history of blood or urine tests among patients with comorbidities that are associated with increased risk for CKD [3, 5, 19, 20]. Blood or urine tests were more frequently recorded in patients with diabetes, hyperuricemia, anemia, heart failure, or hypertension than in patients without these complications (Figs. 4 and 5). However, a certain number of patients with these complications did not have records of blood tests (diabetes: 28.4%; hyperuricemia: 36.8%; anemia: 36.1%; heart failure: 27.5%; hypertension: 41.2%). The majority of patients with complications did not have records of urine tests (diabetes: 62.5%; hyperuricemia: 71.1%; anemia: 73.8%; heart failure: 70.8%; hypertension: 72.5%).

### Incidence of cardiorenal-related events from the index date according to CKD stage at diagnosis

**Fig. 6:**
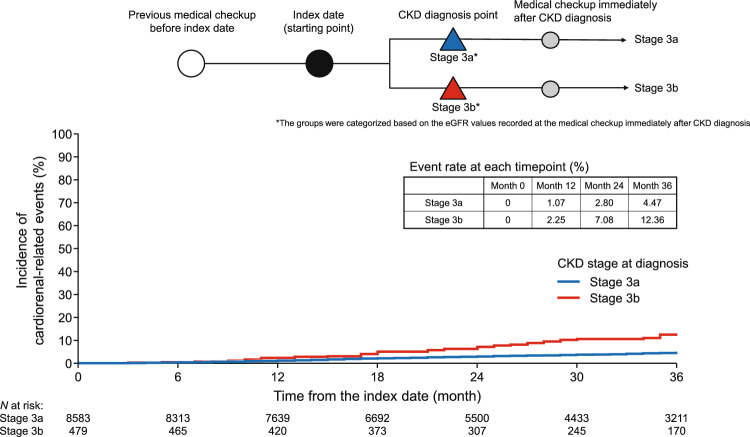
Kaplan–Meier curves of the cumulative incidence of cardiorenal-related events among patients with stage 3a or 3b CKD starting from the index date. The outcome was a composite of chronic hemodialysis, renal transplantation, progression to stage 4/5 CKD, myocardial infarction, stroke, and hospitalization for heart failure, which were identified using appropriate International Classification of Diseases, 10th revision, diagnostic or procedure codes recorded after the diagnosis of stage 3a or 3b CKD. Patients were censored at the first record of a cardiorenal-related event or at the end of the study period for patients without an event. Patients whose insurance was changed during the study period, without a record of a cardiorenal-related event, were excluded from analysis. *CKD* chronic kidney disease, *eGFR* estimated glomerular filtration rate

To identify why early diagnosis is so important in performing blood and urine tests, we investigated the frequency of cardiorenal-related events in patients diagnosed at stage 3a and 3b. The Kaplan–Meier curves for the cumulative proportions of patients with cardiorenal-related events among patients diagnosed at stage 3a (*n* = 8583; eGFR 45 to <60 mL/min/1.73 m^2^) or 3b (*n* = 479; 30 to <45 mL/min/1.73 m^2^) based on renal function at CKD diagnosis are shown in Fig. 6. The incidence of cardiorenal-related events was 1.07% and 2.25% at 12 months, and 4.47% and 12.36% at 36 months for patients diagnosed at stage 3a and 3b, respectively.

Additionally, we reviewed the characteristics of patients diagnosed at stage 3a and 3b, such as the reduction in eGFR and the time to CKD diagnosis, at the time of the index date and the previous medical checkup date before the index date (Table 2). The median eGFR was lower in patients diagnosed at stage 3b (43.90 mL/min/1.73 m^2^) than in patients diagnosed at stage 3a (57.10 mL/min/1.73 m^2^) at the index date. However, at the previous medical checkup date, the median eGFR was similar between the patients diagnosed at stage 3b (65.70 mL/min/1.73 m^2^) and those diagnosed at stage 3a (63.90 mL/min/1.73 m^2^). This result suggests that the reduction in eGFR from the time of diagnosis to the time of the previous medical checkup was larger in patients diagnosed at stage 3b. The interval from the previous medical checkup date before the index date to the index date was similar for stage 3a and 3b (12.0 and 12.0 months, respectively), as was the interval from the index date to the first CKD visit (6.0 and 8.0 months, respectively).

**Table 2:** Characteristics of patients with stage 3a or 3b CKD at the index date^a^

Characteristic	Stage 3a (*N* = 8583)	Stage 3b (*N* = 479)
Sex		
Male	3996 (46.6)	252 (52.6)
Female	4587 (53.4)	227 (47.4)
Age (years)		
<65	3592 (41.9)	133 (27.8)
≥65 to <75	3207 (37.4)	203 (42.4)
≥75	1784 (20.8)	143 (29.9)
Mean ± SD	64.7 ± 12.1	68.7 ± 10.9
Median (Q1–Q3)	67.0 (56.0–72.0)	69.0 (63.0–77.0)
BMI (kg/m^2^)		
<18.5	602 (7.0)	42 (8.8)
≥18.5 to <25.0	5590 (65.2)	293 (61.2)
≥25.0 to <30.0	2042 (23.8)	123 (25.7)
≥30.0	341 (4.0)	21 (4.4)
Unknown	8 (0.1)	0
Mean ± SD	23.21 ± 3.54	23.17 ± 3.73
Median (Q1–Q3)	23.00 (20.80–25.30)	22.90 (20.60–25.50)
History of smoking		
Yes	610 (9.5)	44 (11.3)
No	5815 (90.5)	345 (88.7)
Unknown	2158 (25.1)	90 (18.8)
Complications (in ≥5% of patients)		
Hypertension	3612 (42.1)	277 (57.8)
Dyslipidemia	3503 (40.8)	196 (40.9)
Diabetes	1735 (20.2)	126 (26.3)
Hyperuricemia	812 (9.5)	73 (15.2)
Heart failure	660 (7.7)	73 (15.2)
Ischemic heart disease	640 (7.5)	53 (11.1)
Anemia	633 (7.4)	76 (15.9)
Atrial fibrillation	271 (3.2)	26 (5.4)
History of blood/urine tests		
Blood tests	4728 (55.1)	322 (67.2)
Urine tests	2855 (33.3)	201 (42.0)
Insurer type		
Health insurance	509 (29.2)	85 (17.7)
National Health Insurance	4275 (49.8)	248 (51.8)
Medical insurance system for the elderly	1799 (21.0)	146 (30.5)
eGFR (mL/min/1.73 m^2^) (at a medical checkup prior to index date)		
Mean ± SD	65.36 ± 5.60	70.21 ± 43.48
Median (Q1–Q3)	63.90 (61.80‒67.16)	65.70 (62.30‒71.68)
eGFR (mL/min/1.73 m^2^) (at index date)		
≥90	1 (0.0)	0 (0.0)
≥60 to <90	175 (2.0)	12 (2.5)
≥45 to <60	8373 (97.6)	174 (36.3)
≥30 to <45	29 (0.3)	293 (61.2)
≥15 to <30	0 (0.0)	0 (0.0)
<15	0 (0.0)	0 (0.0)
Unknown	5 (0.1)	0 (0.0)
Mean ± SD	56.34 ± 3.79	45.74 ± 8.63
Median (Q1–Q3)	57.10 (54.64‒58.70)	43.90 (39.47‒53.52)
eGFR (mL/min/1.73 m^2^) (at a medical checkup immediately after CKD diagnosis)		
Mean ± SD	55.64 ± 3.48	40.03 ± 3.90
Median (Q1–Q3)	56.60 (53.80‒58.40)	40.90 (37.40‒43.30)
Urine protein		
−	6852 (80.1)	334 (69.9)
±	954 (11.1)	58 (12.1)
1+	569 (6.6)	49 (10.3)
2+	158 (1.8)	25 (5.2)
3+	24 (0.3)	12 (2.5)
4+	0 (0.0)	0 (0.0)
Unknown	26 (0.3)	1 (0.2)
Interval between the prior medical checkup and the index date (months)		
Median (Q1–Q3)	12.0 (11.0–14.0)	12.0 (12.0–14.0)
Interval between the index date and first CKD diagnosis (months)		
Median (Q1–Q3)	6.0 (2.0–14.0)	8.0 (2.0–17.0)

Values are *n* (%) unless otherwise specified

*BMI* body mass index, *CKD* chronic kidney disease, *eGFR* estimated glomerular filtration rate, *Q* quartile, *SD* standard deviation

^a^Patient characteristics were retrieved from the medical records at, or within 12 months before, index date

Finally, we determined the incidence of cardiorenal-related events according to the reduction in eGFR over time. As shown in Fig. S1 (Online Resource 1), the cumulative incidence was numerically greatest (6.66%) in patients with the numerically largest change in eGFR (<−15.0 mL/min/1.73 m^2^).

## Discussion

This study clarified the current status regarding CKD diagnosis in Japan as well as the prognostic impact of stage at diagnosis, considering recent changes to the treatment landscape. With the increased importance of early CKD diagnosis by the advancements of CKD Clinical Practice Guidelines and the launch of SGLT2 inhibitors, we anticipated that the rate of CKD diagnosis would increase. However, the CKD diagnosis rate remained stable: 2.6% to 3.5% in 2014–2016 before the revisions of the CKD Clinical Practice Guidelines and 3.0% to 3.4% in 2017 even after the revision. The REVEAL-CKD study, which used a Japanese real-world database [21], also showed that the percentage of patients diagnosed with CKD at stage 3 at medical checkups was 7.9%, and the percentage of patients not diagnosed with CKD was ≥90%. There are several reasons for the low CKD diagnosis rate. First, the data used in this study depend on CKD diagnosis codes based on insurance claims data, and there may be cases in which CKD diagnosis names are not added, which may underestimate the CKD diagnosis rate compared to actual clinical practice. This may be the case, for example, when renin-angiotensin system (RAS) inhibitors are already used in patients with a diagnosis of hypertension. Another possible reason is the low frequency of testing renal function in actual clinical practice. Since CKD is only clinically diagnosed when a patient has eGFR <60 mL/min/1.73 m^2^ and/or urine protein ≥1+ for ≥3 months, patients with one test satisfying either of these criteria should undergo a follow-up blood/urine test or periodic testing to confirm the diagnosis of CKD. Indeed, as shown in Fig. 3, patients with possible CKD who underwent blood/urine testing at medical institutions within 6 months after the index date were more likely to be diagnosed with CKD than those who did not.

Furthermore, we found that large proportions of patients with comorbidities, such as diabetes, hyperuricemia, anemia, or heart failure, did not undergo blood tests (Fig. 4) or urine tests (Fig. 5), even though patients with these comorbidities were more likely to be diagnosed with CKD than patients without these comorbidities (Fig. 3). Patients with these comorbidities are often diagnosed with CKD [21] and these comorbidities are well-known risk factors for CKD. As such, the guidelines for CKD emphasize the importance of renal function testing for patients with these comorbidities [3, 4, 10]. It is preferable that patients with possible CKD based on a medical checkup undergo blood/urine tests at a medical institution, but our results indicate that blood/urine tests are not being sufficiently performed at medical institutions, even for patients with comorbidities. Regular blood/urine testing is valuable for the swift diagnosis of various clinically significant pathologies, not just CKD [3, 4, 22]. By shedding light on a significant gap in clinical practice, namely inadequate performance of diagnostic testing in patients with clinical risk factors for CKD, these results should help to raise awareness of this problem for healthcare professionals and could contribute to the timely diagnosis and treatment of CKD in the future. Patients at risk of showing signs of renal impairment based on eGFR or urinary protein levels assessed at medical checkups should be actively considered for additional renal function tests at the time of their medical visit. Furthermore, we believe it is important for these patients to visit a medical institution for diagnosis and to initiate appropriate treatment.

When comparing the prognosis of patients according to renal function (i.e., CKD stage), the incidence of cardiorenal-related events is higher in patients with more advanced stages of CKD [21]. However, it is not known whether different diagnostic stages influence prognosis when the conditions are the same at a starting point. Therefore, we compared the prognosis of patients with the same condition, i.e., a condition that was first found to have the potential for CKD at physical examination, as a starting point (Fig. 6). Even over 36 months following the medical examination that first met the CKD diagnostic criteria, the cumulative incidence of cardiorenal-related events was notably higher in patients diagnosed at stage 3b than in those diagnosed at stage 3a. Therefore, considering the incidence of cardiorenal-related events in stage 3a and 3b, diagnosis at an early stage (stage 3a) is preferable.

This difference in the cumulative incidence of cardiorenal-related events could be attributed to differences in the baseline condition and/or the interval from the start of observation to CKD diagnosis. Interestingly, the median interval between the medical examination and CKD diagnosis was comparable between patients diagnosed at stage 3a and 3b (6.0 and 8.0 months, respectively; Table 2), suggesting that the observation-to-diagnosis period did not influence the stage of diagnosis. Likewise, the median interval between medical checkups was similar between these groups (12.0 and 12.0 months, respectively). However, the median eGFR at the start of observation was lower in patients diagnosed at stage 3b (43.90 mL/min/1.73 m^2^) than in those diagnosed at stage 3a (57.10 mL/min/1.73 m^2^), indicating a more advanced state of CKD at the initial observation in patients diagnosed at stage 3b. The median eGFR at the preceding medical examination did not differ substantially between stage 3a (63.90 mL/min/1.73 m^2^) and 3b (65.70 mL/min/1.73 m^2^). These findings suggest that individuals diagnosed at stage 3b experienced a more pronounced decline in eGFR within about 1 year between medical checkups. This led us to postulate that the magnitude of decline in eGFR during this period may account for the observed variation in cardiorenal-related event rates between the two stages. This theory is corroborated by Fig. S1 (Online Resource 1), which demonstrates that patients with greater annualized decline in eGFR had higher rates of cardiorenal-related events [23]. Furthermore, a systematic review confirmed the strong correlation between rapid eGFR deterioration and cardiorenal-related events in patients with type 2 diabetes [24]. Therefore, because a rapid decrease in eGFR may indicate future risk of a cardiorenal-related event, it is important to monitor the change in eGFR in such patients, not just those with an eGFR <60 mL/min/1.73 m^2^. For this reason, we recommend periodic renal function tests, which may lead to detection of CKD in an early stage.

This study has several limitations that may contribute to bias. First, because the data used in this study rely on the CKD diagnosis code recorded in the insurance claim data, the CKD diagnosis rate may be underestimated relative to the actual rate in clinical practice. For example, if RAS inhibitors have already been used in the diagnosis of hypertension, the term for CKD diagnosis may not be added to the claims data. Moreover, the study period after the approval of SGLT2 inhibitors for CKD was only 1 year. A longer study period will be needed to investigate the effects of greater knowledge and use of SGLT2 inhibitors in the context of diagnosis and treatment of CKD in clinical practice. Second, this study involved retrospective analyses of a database that accumulated data from three independent sources (Society-Managed Health Insurance, National Health Insurance managed by municipalities, and the Medical Care System for the Elderly). Although the multiple sources of data used here could be an advantage, particularly the inclusion of younger and older patients to better reflect the Japanese population, some patients may receive medical care through different systems, including the Japan Health Insurance Association and Mutual Aid Association. In addition, the three data sources are complete and independent units, and it is not possible to link data across different health insurance systems, limiting traceability. Nevertheless, patients captured within the DeSC database can be continuously followed up, even across different medical institutions, until a change in health insurance association. Third, medical checkups were the only source of clinical outcome tests in this study. Medical checkups are typically performed annually, and there may be missing data or data entry errors. Additionally, even if a large decrease in eGFR is confirmed at a medical institution examination after a medical checkup (i.e., index date), it cannot be confirmed in the current analysis. In addition, the analysis was limited to patients who underwent medical checkups, who may be healthier than average Japanese individuals, leading to selection bias.

In conclusion, the prevalence of CKD diagnosis in Japan was low and renal function tests were not sufficiently performed. Furthermore, the correlation between blood/urine testing and CKD diagnosis at medical institutions, and the incidence of cardiorenal events, were lower in patients diagnosed with CKD in stage 3a than in stage 3b. These findings suggest that regular blood/urine testing may be useful for early detection of CKD.

## Supplementary Information

Below is the link to the electronic supplementary material.

Supplementary file1 (DOCX 172 KB)

## Data Availability

The data that support the findings of this study are available from DeSC Healthcare Inc.™. but restrictions apply to the availability of these data, which were used under license for the current study, and so are not publicly available. Data are however available from the authors upon reasonable request and with permission of DeSC Healthcare Inc.™.
