# Supplementary material for: The diagnostic status of chronic kidney disease in a real-world database in Japan: CHECK-CKD
Source: Clin Exp Nephrol. 2025 May 27;29(9):1212–22. doi: 10.1007/s10157-025-02682-z (PMC12441055; doi:10.1007/s10157-025-02682-z)

# Electronic Supplementary materials: Online resource 1

# The diagnostic status of chronic kidney disease in a real-world database in Japan: CHECK-CKD

Toshiki Moriyama, Keigo Kanafuri, Mayu Kanno, Koji Niibe, Sachiko Nago, Ichiro Fukuoka, Yasuhisa Fukunaga, Issei Doi, Masaru Kawashima

**Corresponding author:** Toshiki Moriyama

**Email:** toshiki.moriyama@gmail.com

| **Content** | **Page** |
| --- | --- |
| **Table S1.** Diagnostic and procedure codes | 2 |
| **Table S2.** Characteristics of the matched cohorts of patients with and without CKD diagnosis at, or within 12 months before, the matching timepoint | 3 |
| **Table S3.** Characteristics of patients without history of CKD who satisfied the CKD criteria (eGFR <60 mL/min/1.73 m^2^ or urine protein ≥1+) between August 2021 and July 2022 according to history of blood tests at a medical institution | 5 |
| **Table S4.** Characteristics of patients who underwent medical checkups between August 2021 and July 2022 according to history of urine tests at a medical institution | 7 |
| **Figure S1.** Kaplan–Meier curves of the cumulative incidence of cardiorenal events among patients stratified according to the annual decline in eGFR | 9–10 |

**Table S1** Diagnostic and procedure codes

| **Diagnosis** | **ICD-10/procedure codes** |
| --- | --- |
| CKD | E102, E112, E132, E142, I120, I129, I139, I151, N028‒N030, N032‒N034, N036, N037, N039‒N044, N046, N048‒N059, N069, N110, N111, N118, N119, N12, N130‒N133, N140‒N144, N151, N159, N181‒N185, N189, N19, N209, N250, N251, N258, N259, N26, N280, N281, N288, N289, N391, Q611‒Q613, Q618 |
| Anemia | D461, D462, D464, D500, D508‒D513, D518‒D521, D528‒D532, D538, D539, D550‒D552, D559‒D561, D563, D564, D569, D580‒D582, D588‒D592, D594, D599, D610‒D613, D619, D62, D640‒D644, D648, D649, E039, E230, E274, H350, K769, N19, O990, P612, P614 |
| Diabetes | E10, E100‒E107, E109, E11, E110‒E116, E12, E13, E130‒E137, E139, E14, E140‒E146, E149, E831, E881, E888, E891, O240, O241, O244, O249, P701, P702 |
| Hypertension | I10, I110, I119, I120, I129, I139, I150‒I152, I159, I270, I674, O100‒O102, O104, O11, O13, O140, O141, O149, O16, O94, P000, P292, P293, R030 |
| Dyslipidemia | E106, E116, E780‒E782, E785, E789 |
| Hyperuricemia | E790 |
| Heart failure | I110, I500, I501, I509, O291 |
| Stroke | G459, G819, I600‒I611, I613‒I616, I618, I619, I629‒I636, I638, I639, I64, I660‒I663, I668, I669, I690, I691, I693, I694, P103, S066, S0660, S0661 |
| Atrial fibrillation | I480 |
| Ischemic heart disease | I248, I249, I255, I256, I258, I259 |
| NASH/NAFLD | K758, K760 |
| COPD | J440, J441, J448, J449 |
| Chronic dialysis | 113002510, 114003510, 114003610, 114006610, 114009310, 114009410, 114009510, 114061370, 140007710, 140007910, 140008170, 140008510, 140008770, 140008810, 140029850, 140033770, 140036710, 140051010, 140051110, 140052570, 140052810, 140052970, 140053670, 140054850, 140054950, 140055970, 140057810, 140057910, 140058010, 140058110, 140058210, 140058310, 140058410, 140058510, 140058610, 140058770, 140058870, 140058970, 140059070, 140059170, 140059270, 140059310, 140059410, 140059510, 140060210, 140060310, 140060410, 140060510, 140060610, 140060710, 140060810, 140060910, 140061010, 140062770, 140062870 |
| Kidney transplantation | T861, Z940, 150196310, 150196410, 150196570, 150338610, 150420970, 150421070 |
| CKD stage 4 or 5 | N184, N185 |
| Myocardial infarction | I210‒I214, I219‒I221, I228‒I236, I238, I241, I252 |
| Urine tests^a^ | 160000310, 160000410, 160004810 |
| Blood tests | 160019210 |

*CKD* chronic kidney disease, *COPD* chronic obstructive pulmonary disease, *ICD-10* International Classification of Diseases, 10th revision, *NAFLD* non-alcoholic fatty liver disease, *NASH* non-alcoholic steatohepatitis

^a^Urine tests: urine general, urine protein, or urine albumin assay

**Table S2** Characteristics of the matched cohorts of patients with and without CKD diagnosis at, or within 12 months before, the matching timepoint^a^

| **Characteristic** |  | **Patients with  CKD diagnosis**  **(*N* = 22,207)** | **Patients without  CKD diagnosis**  **(*N* = 22,207)** |
| --- | --- | --- | --- |
| Sex | Male | 11,422 (51.4) | 10,145 (45.7) |
|  | Female | 10,785 (48.6) | 12,062 (54.3) |
| Age (years) | <65 | 7947 (35.8) | 7437 (33.5) |
|  | ≥65 to <75 | 9020 (40.6) | 10,253 (46.2) |
|  | ≥75 | 5240 (23.6) | 4517 (20.3) |
|  | Mean ± SD | 66.4 ± 13.1 | 66.3 ± 12.3 |
|  | Median (Q1–Q3) | 69.0 (57.0–74.0) | 69.0 (59.0–73.0) |
| BMI (kg/m^2^) | <18.5 | 1396 (7.3) | 1298 (6.7) |
|  | ≥18.5 to <25.0 | 11,854 (61.6) | 12,152 (63.1) |
|  | ≥25.0 to <30.0 | 4846 (25.2) | 4795 (24.9) |
|  | ≥30.0 | 1140 (5.9) | 1007 (5.2) |
|  | Unknown | 2971 (13.4) | 2955 (13.3) |
|  | Mean ± SD | 23.53 ± 3.90 | 23.47 ± 3.76 |
|  | Median (Q1–Q3) | 23.20 (20.90–25.70) | 23.10 (20.90–25.50) |
| History of smoking | Yes | 1592 (11.8) | 1517 (10.9) |
|  | No | 11,904 (88.2) | 12,391 (89.1) |
|  | Unknown | 8711 (39.2) | 8299 (37.4) |
| Complications (in ≥5% of patients) | Hypertension | 12,305 (55.4) | 11,468 (51.6) |
|  | Dyslipidemia | 12,262 (55.2) | 10,709 (48.2) |
|  | Diabetes | 7706 (34.7) | 4985 (22.4) |
|  | Heart failure | 3555 (16.0) | 2058 (9.3) |
|  | Hyperuricemia | 3405 (15.3) | 2050 (9.2) |
|  | Anemia | 3204 (14.4) | 1516 (6.8) |
|  | Ischemic heart disease | 2840 (12.8) | 2188 (9.9) |
|  | Stroke | 2298 (10.3) | 1785 (8.0) |
|  | Atrial fibrillation | 1170 (5.3) | 854 (3.8) |
| History of blood/urine tests | Blood tests | 19,266 (86.8) | 13,643 (61.4) |
|  | Urine tests | 15,517 (69.9) | 7022 (31.6) |
| Insurer type | Health insurance | 5906 (26.6) | 5357 (24.1) |
|  | National Health Insurance | 11,027 (49.7) | 12,326 (55.5) |
|  | Medical insurance system for the elderly | 5274 (23.7) | 4524 (20.4) |
| eGFR (mL/min/1.73 m^2^) | Stage 1: ≥90 | 804 (4.2) | 858 (4.5) |
|  | Stage 2: ≥60 to <90 | 6299 (33.3) | 7328 (38.7) |
|  | Stage 3a: ≥45 to <60 | 11,009 (58.1) | 10,607 (56.0) |
|  | Stage 3b: ≥30 to <45 | 712 (3.8) | 138 (0.7) |
|  | Stage 4: ≥15 to <30 | 95 (0.5) | 4 (0.0) |
|  | Stage 5: <15 | 20 (0.1) | 15 (0.1) |
|  | Unknown | 3268 (14.7) | 3257 (14.7) |
|  | Mean ± SD | 61.56 ± 13.37 | 63.67 ± 12.36 |
|  | Median (Q1–Q3) | 58.54 (54.86–65.80) | 59.37 (56.80–67.60) |
| Urine protein | − | 12,100 (63.1) | 13,510 (70.4) |
|  | ± | 2032 (10.6) | 1788 (9.3) |
|  | 1+ | 3736 (19.5) | 3389 (17.7) |
|  | 2+ | 1034 (5.4) | 431 (2.2) |
|  | 3+ | 277 (1.4) | 72 (0.4) |
|  | 4+ | 3 (0.0) | 0 (0.0) |
|  | Unknown | 3025 (13.6) | 3017 (13.6) |

Values are *n* (%) unless otherwise specified

*BMI* body mass index, *CI* confidence interval, *CKD* chronic kidney disease, *eGFR* estimated glomerular filtration rate, *Q* quartile, *SD* standard deviation

^a^Patient characteristics were retrieved from the medical records at, or within 12 months before, the matching timepoint

Patients with and without CKD were matched at the ratio of 1:1 without replacement at each timepoint of the observation period. Patients without CKD were randomly selected from the at-risk population from the period corresponding to patients with CKD. The characteristics of the patients were extracted and multivariable logistic regression was performed to determine the odds ratio and 95% CI for CKD diagnosis, which was identified using relevant International Classification of Disease, 10th revision, diagnostic or procedure codes. The observation items included sex, age (years), BMI (kg/m^2^), smoking history, complications, blood test history, urinalysis history, category of insurer, department of consultation, region of the consultation facility, hospital category (number of beds), eGFR (mL/min/1.73 m^2^), and urinary protein.

**Table S3** Characteristics of patients without history of CKD who satisfied the CKD criteria (eGFR <60 mL/min/1.73 m^2^ and/or urine protein ≥1+) between August 2021 and July 2022 according to history of blood tests at a medical institution^a,b,c^

| **Characteristc** |  | **All patients**  **(*N* = 43,688)** | **Patients with history of blood tests at a medical institution**  **(*N* = 18,912)** | **Patients without history of blood tests at a medical institution**  **(*N* = 24,776)** |
| --- | --- | --- | --- | --- |
| Sex | Male | 22,629 (51.8) | 9900 (52.3) | 12,729 (51.4) |
|  | Female | 21,059 (48.2) | 9012 (47.7) | 12,047 (48.6) |
| Age (years) | <65 | 24,912 (57.0) | 8745 (46.2) | 16,167 (65.3) |
|  | 65 to <75 | 13,076 (29.9) | 6734 (35.6) | 6342 (25.6) |
|  | ≥75 | 5700 (13.0) | 3433 (18.2) | 2267 (9.1) |
|  | Mean ± SD | 60.4 ± 13.6 | 64.0 ± 12.7 | 57.7 ± 13.7 |
|  | Median (Q1–Q3) | 61.0 (51.0‒71.0) | 66.0 (54.0‒73.0) | 57.0 (49.0‒69.0) |
| BMI (kg/m^2^) | <18.5 | 2906 (6.7) | 1084 (5.7) | 1822 (7.4) |
|  | 18.5 to <25.0 | 27,022 (61.9) | 11,285 (59.7) | 15,737 (63.5) |
|  | 25.0 to <30.0 | 11,122 (25.5) | 5199 (27.5) | 5923 (23.9) |
|  | ≥30.0 | 2636 (6.0) | 1342 (7.1) | 1294 (5.2) |
|  | Unknown | 2 (0.0) | 2 (0.0) | 0 (0.0) |
|  | Mean ± SD | 23.61 ± 3.89 | 23.94 ± 3.98 | 23.35 ± 3.80 |
|  | Median (Q1–Q3) | 23.20 (21.00‒25.70) | 23.60 (21.30‒26.10) | 23.00 (20.70‒25.40) |
| History of smoking | Yes | 2010 (10.3) | 901 (9.4) | 1109 (11.2) |
|  | No | 17,437 (89.7) | 8634 (90.6) | 8803 (88.8) |
|  | Unknown | 24,241 (55.5) | 9377 (49.6) | 14,864 (60.0) |
| Complications (in ≥5% of patients) | Hypertension | 16,640 (38.1) | 9789 (51.8) | 6851 (27.7) |
|  | Dyslipidemia | 16,282 (37.3) | 10,090 (53.4) | 6192 (25.0) |
|  | Diabetes | 7461 (17.1) | 5342 (28.2) | 2119 (8.6) |
|  | Hyperuricemia | 4035 (9.2) | 2550 (13.5) | 1485 (6.0) |
|  | Ischemic heart disease | 2986 (6.8) | 2030 (10.7) | 956 (3.9) |
|  | Heart failure | 2739 (6.3) | 1985 (10.5) | 754 (3.0) |
|  | Stroke | 2205 (5.0) | 1396 (7.4) | 809 (3.3) |
|  | Anemia | 2523 (5.8) | 1612 (8.5) | 911 (3.7) |
| Insurer type | Health insurance | 24,191 (55.4) | 8593 (45.4) | 15,598 (63.0) |
|  | National Health Insurance | 13,787 (31.6) | 6869 (36.3) | 6918 (27.9) |
|  | Medical insurance system for the elderly | 5710 (13.1) | 3450 (18.2) | 2260 (9.1) |
| eGFR (mL/min/1.73 m^2^) | Stage 1: ≥90 | 1668 (3.8) | 553 (2.9) | 1115 (4.5) |
|  | Stage 2: ≥60 to <90 | 7411 (17.1) | 2948 (15.7) | 4463 (18.1) |
|  | Stage 3a: ≥45 to <60 | 33,783 (77.8) | 14,891 (79.2) | 18,892 (76.7) |
|  | Stage 3b: ≥30 to <45 | 543 (1.3) | 386 (2.1) | 157 (0.6) |
|  | Stage 4: ≥15 to <30 | 14 (0.0) | 14 (0.1) | 0 (0.0) |
|  | Stage 5: <15 | 14 (0.0) | 8 (0.0) | 6 (0.0) |
|  | Unknown | 255 (0.6) | 112 (0.6) | 143 (0.6) |
|  | Mean ± SD | 60.79 ± 14.62 | 59.65 ± 12.70 | 61.66 ± 15.87 |
|  | Median (Q1–Q3) | 58.00 (55.32‒59.77) | 57.66 (54.80‒59.50) | 58.22 (55.80‒59.84) |
| Urine protein | − | 29,775 (68.4) | 13,021 (69.1) | 16,754 (67.8) |
|  | ± | 3151 (7.2) | 1507 (8.0) | 1644 (6.7) |
|  | 1+ | 9239 (21.2) | 3634 (19.3) | 5605 (22.7) |
|  | 2+ | 1203 (2.8) | 591 (3.1) | 612 (2.5) |
|  | 3+ | 191 (0.4) | 98 (0.5) | 93 (0.4) |
|  | 4+ | 1 (0.0) | 1 (0.0) | 0 (0.0) |
|  | Unknown | 128 (0.3) | 60 (0.3) | 68 (0.3) |

Values are *n* (%) unless otherwise specified

*BMI* body mass index, *CI* confidence interval, *CKD* chronic kidney disease, *eGFR* estimated glomerular filtration rate, *Q* quartile, *SD* standard deviation

^a^Eligible patients were those with a medical examination satisfying CKD criteria between August 2021 and July 2022 before the diagnosis of CKD and were followed up for 6 months from the examination and had a medical institution visit within 6 months

^b^Patient characteristics were retrieved from the medical records at, or within 12 months before, the medical checkup date

^c^History of blood tests was defined as the presence or absence of codes for blood tests in patient records

**Table S4** Characteristics of patients who underwent medical checkups between August 2021 and July 2022 according to history of urine tests at a medical institution^a,b,c^

| **Characteristic** |  | **All patients**  **(*N* = 43,688)** | **Patients with history of urine tests at a medical institution**  **(*N* = 9110)** | **Patients without history of urine tests at a medical institution**  **(*N* = 34,578)** |
| --- | --- | --- | --- | --- |
| Sex | Male | 22,629 (51.8) | 4908 (53.9) | 17,721 (51.2) |
|  | Female | 21,059 (48.2) | 4202 (46.1) | 16,857 (48.8) |
| Age (years) | <65 | 24,911 (57.0) | 4205 (46.2) | 20,706 (59.9) |
|  | 65 to <75 | 13,077 (29.9) | 3225 (35.4) | 9852 (28.5) |
|  | ≥75 | 5700 (13.0) | 1680 (18.4) | 4020 (11.6) |
|  | Mean ± SD | 60.4 ± 13.6 | 63.4 ± 13.6 | 59.6 ± 13.5 |
|  | Median (Q1–Q3) | 61.0 (51.0‒71.0) | 66.0 (54.0‒73.0) | 59.0 (50.0‒71.0) |
| BMI (kg/m^2^) | <18.5 | 2903 (6.6) | 578 (6.3) | 2325 (6.7) |
|  | 18.5 to <25.0 | 27,020 (61.9) | 5416 (59.5) | 21,604 (62.5) |
|  | 25.0 to <30.0 | 11,127 (25.5) | 2461 (27.0) | 8666 (25.1) |
|  | ≥30.0 | 2636 (6.0) | 655 (7.2) | 1981 (5.7) |
|  | Unknown | 2 (0.0) | 0 (0.0) | 2 (0.0) |
|  | Mean ± SD | 23.61 ± 3.89 | 23.90 ± 4.07 | 23.53 ± 3.84 |
|  | Median (Q1–Q3) | 23.20 (21.00‒25.70) | 23.50 (21.20‒26.00) | 23.20 (20.90‒25.60) |
| History of smoking | Yes | 2011 (10.3) | 459 (10.0) | 1552 (10.4) |
|  | No | 17,436 (89.7) | 4130 (90.0) | 13,306 (89.6) |
|  | Unknown | 24,241 (55.5) | 4521 (49.6) | 19,720 (57.0) |
| Complications (in ≥5% of patients) | Hypertension | 16,640 (38.1) | 4582 (50.3) | 12,058 (34.9) |
|  | Dyslipidemia | 16,281 (37.3) | 4605 (50.5) | 11,676 (33.8) |
|  | Diabetes | 7460 (17.1) | 2796 (30.7) | 4664 (13.5) |
|  | Hyperuricemia | 4035 (9.2) | 1166 (12.8) | 2869 (8.3) |
|  | Ischemic heart disease | 2985 (6.8) | 874 (9.6) | 2111 (6.1) |
|  | Heart failure | 2740 (6.3) | 800 (8.8) | 1940 (5.6) |
|  | Stroke | 2206 (5.0) | 628 (6.9) | 1578 (4.6) |
|  | Anemia | 2521 (5.8) | 661 (7.3) | 1860 (5.4) |
| Insurer type | Health insurance | 24,191 (55.4) | 4093 (44.9) | 20,098 (58.1) |
|  | National Health Insurance | 13,787 (31.6) | 3330 (36.6) | 10,457 (30.2) |
|  | Medical insurance system for the elderly | 5710 (13.1) | 1687 (18.5) | 4023 (11.6) |
| eGFR (mL/min/1.73 m^2^) | Stage 1: ≥90 | 1669 (3.8) | 413 (4.6) | 1256 (3.7) |
|  | Stage 2: ≥60 to <90 | 7410 (17.1) | 1905 (21.1) | 5505 (16.0) |
|  | Stage 3a: ≥45 to <60 | 33,783 (77.8) | 6537 (72.3) | 27,246 (79.2) |
|  | Stage 3b: ≥30 to <45 | 543 (1.3) | 176 (1.9) | 367 (1.1) |
|  | Stage 4: ≥15 to <30 | 14 (0.0) | 4 (0.0) | 10 (0.0) |
|  | Stage 5: <15 | 14 (0.0) | 4 (0.0) | 10 (0.0) |
|  | Unknown | 255 (0.6) | 71 (0.8) | 184 (0.5) |
|  | Mean ± SD | 60.79 ± 14.62 | 61.42 ± 15.38 | 60.62 ± 14.41 |
|  | Median (Q1–Q3) | 58.00 (55.32‒59.77) | 58.00 (55.00‒60.95) | 58.00 (55.40‒59.70) |
| Urine protein | − | 29,773 (68.3) | 5553 (61.1) | 24,220 (70.2) |
|  | ± | 3153 (7.2) | 717 (7.9) | 2436 (7.1) |
|  | 1+ | 9238 (21.2) | 2271 (25.0) | 6967 (20.2) |
|  | 2+ | 1204 (2.8) | 454 (5.0) | 750 (2.2) |
|  | 3+ | 191 (0.4) | 86 (0.9) | 105 (0.3) |
|  | 4+ | 1 (0.0) | 1 (0.0) | 0 (0.0) |
|  | Unknown | 128 (0.3) | 28 (0.3) | 100 (0.3) |

Values are *n* (%) unless otherwise specified

*BMI* body mass index, *CKD* chronic kidney disease, *eGFR* estimated glomerular filtration rate, *Q* quartile, *SD* standard deviation

^a^Eligible patients were those with a medical examination satisfying CKD criteria between August 2021 and July 2022 before the diagnosis of CKD and were followed up for 6 months from the examination and had a medical institution visit within 6 months

^b^Patient characteristics were retrieved from the medical records at, or within 12 months before, the medical checkup date

^c^History of urine tests was defined as the presence or absence of codes for urine tests in patient records

**Figure S1.** Kaplan–Meier curves of the cumulative incidence of cardiorenal events among patients stratified according to the annual decline in eGFR. The annual decline in eGFR was calculated as the difference in eGFR between (i) the medical checkup prior to the index date and (ii) the medical checkup satisfying the criteria for CKD (i.e., index date), and was categorized into the following five levels: <−15.0, −15.0 to <−10.0, −10.0 to < −5.0, −5.0 to <0.0, and ≥0 mL/min/1.73 m^2^. As above, patients were censored at the time of the first cardiorenal event or at the end of the study period for patients without an event. The outcome was a composite of chronic hemodialysis, renal transplantation, progression to stage 4/5 CKD, myocardial infarction, stroke, and hospitalization for heart failure, which were identified using relevant International Classification of Diseases, 10th revision, diagnostic or procedure codes. *CKD* chronic kidney disease, *eGFR* estimated glomerular filtration rate


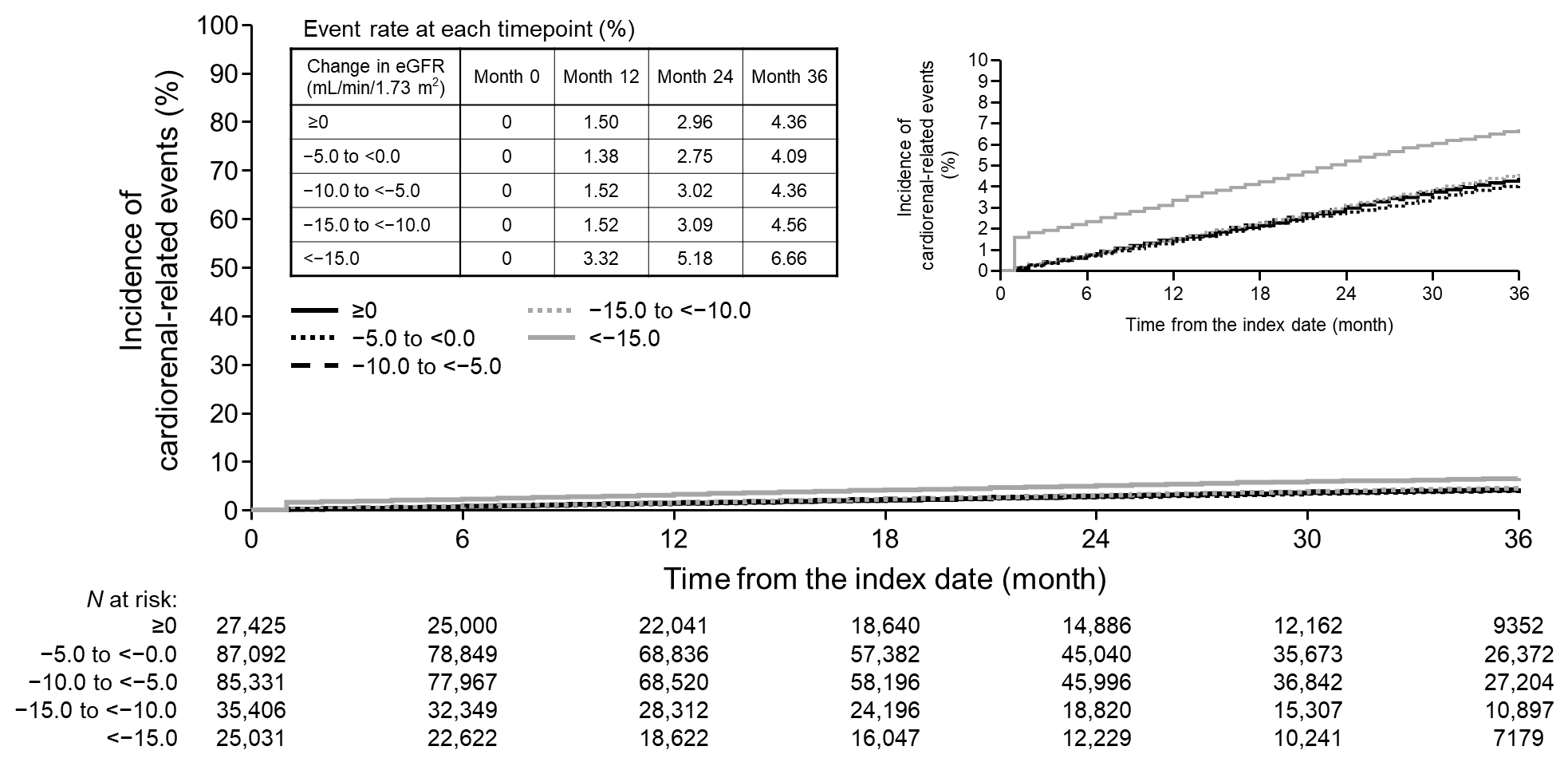

Supplement: Supplementary file 1 — Supplementary file1 (DOCX 172 KB) [file 10157_2025_2682_MOESM1_ESM.docx]
